# Dermatological Manifestations of Smoking-Induced Oxidative Stress and Inflammation: A Multifaceted Analysis of Cutaneous Aging and Disease Progression

**DOI:** 10.7759/cureus.94147

**Published:** 2025-10-08

**Authors:** Jazba Yousaf, Syeda Sakina, Afshan Saeed, Maria Aftab, Muhammad Iftikhar Khattak, Abdullah Elrefae, Miqdad Qandeel, Awais Hameed

**Affiliations:** 1 Department of Geriatric Medicine, Russells Hall Hospital, Dudley, GBR; 2 Department of Dermatology, Dr. Sakina Clinic, Wah Cantt, PAK; 3 Department of Dermatology, Ashford and St Peter's Hospital, Ashford, GBR; 4 Department of Geriatrics, Royal Infirmary of Edinburgh, Edinburgh, GBR; 5 Department of Research and Development, Celestial and Dimanche, Muzaffarabad, PAK; 6 Department of Trauma and Orthopaedic, AlBashir Hospital, Amman, JOR; 7 Department of Trauma and Orthopaedics, Central Middlesex Hospital, London, GBR; 8 Department of Bioinformatics and Biotechnology, Government College University Faisalabad, Faisalabad, PAK

**Keywords:** cutaneous aging, dermatology, inflammation, machine learning, oxidative stress, psoriasis, skin cancer, smoking

## Abstract

Background

Smoking is a leading preventable cause of morbidity and mortality, with significant yet underrecognized dermatological consequences. Tobacco-induced oxidative stress and inflammation contribute to cutaneous aging, chronic inflammatory dermatoses, and carcinogenesis.

Objective

This study aimed to investigate dermatological manifestations associated with smoking, focusing on clinical outcomes, laboratory biomarkers, and predictive modeling using machine learning.

Methods

A retrospective dataset of 350 patients was analyzed. Demographic, clinical, and laboratory variables were assessed using chi-square tests, analysis of variance (ANOVA), t-tests, and correlation analyses. Machine learning models (Random Forest, Logistic Regression, (XGBoost Developers, Distributed Machine Learning Community, Washington, DC), and LightGBM (Microsoft Corporation, Redmond, WA)) were applied to predict psoriasis presence; however, all demonstrated poor discriminatory performance (area under the curve (AUC) < 0.50). Despite limited accuracy, feature importance analysis highlighted CRP, IL-6, pack-years, and age as relevant contributors, underscoring the methodological challenges of prediction using a synthetic dataset.

Results

The cohort included 51.1% males and 48.9% females, with a mean age of 51.4 years. Current smokers comprised 46.9% of patients and had significantly higher pack-year exposure than former smokers (t = 2.53, p = 0.013). Smoking status was significantly associated with psoriasis prevalence (χ² = 21.38, p < 0.001), atopic dermatitis (χ² = 14.27, p < 0.001), advanced cutaneous aging (χ² = 12.61, p < 0.001), and skin cancer risk (χ² = 18.64, p < 0.001). No significant associations were observed with acne severity, treatment type, or comorbidities. Machine learning models showed poor predictive ability (AUC < 0.50), though feature importance identified CRP, IL-6, pack-years, and age as major contributors.

Conclusion

Smoking is significantly associated with multiple dermatological outcomes, emphasizing the need to integrate smoking history into dermatological evaluation and counseling.

## Introduction

Smoking has accounted for more than 175 million deaths globally over the past three decades [1]. Despite substantial progress in reducing smoking prevalence in many countries, smoking remains a leading risk factor for preventable morbidity and mortality [2]. More than one in ten global deaths and nearly 142 million years of life lost (YLLs) were attributable to smoking in 2021 [1]. Smoking also has important effects on healthcare costs, productivity, and health disparities [3-5]. As a result, tobacco control is an enduring policy and public health priority, with enormous potential to improve population health.

Cigarette smoke contains over 4,000 chemicals, including a high concentration of reactive oxygen species (ROS), free radicals (free radicals are unstable, highly reactive molecules generated during smoking that cause oxidative damage to lipids, proteins, and DNA, thereby accelerating cutaneous aging and inflammation), and other xenobiotics that disrupt skin homeostasis [5]. These compounds generate oxidative stress, impair mitochondrial function, and initiate lipid peroxidation, cascading into DNA damage and premature cellular senescence. Notably, smoking activates matrix metalloproteinases (MMP-1 and MMP-9), which degrade collagen and elastin, contributing to structural deterioration of the dermis and early onset of skin aging [6,7].

The term "smoker's face" reflects a recognizable phenotype, characterized by deep perioral wrinkles, sagging skin, pallor, and uneven pigmentation. Twin studies have illustrated that long-term smokers exhibit significantly more facial aging compared to their non-smoking counterparts, independent of sun exposure [8,9]. These phenotypic changes are not merely cosmetic; they often signify deeper pathological processes tied to chronic inflammation and immune dysregulation.

Inflammatory pathways also play a central role in smoking-related dermatopathology, as tobacco exposure activates nuclear factor kappa B (NF-κB), which then translocates to the nucleus and promotes secretion of pro-inflammatory cytokines such as IL-6, TNF-α, and CRP, thereby impairing epidermal integrity, delaying wound healing, and driving chronic skin inflammation. These mediators compromise epidermal integrity, impair wound healing, and predispose the skin to chronic inflammatory dermatoses [10,11]. Epidemiological data suggest that smokers are up to twice as likely to develop psoriasis as non-smokers, with disease severity often correlating with smoking intensity and duration. Similarly, smoking has been implicated in the exacerbation of acne, hidradenitis suppurativa, and atopic dermatitis, reflecting its widespread impact on inflammatory skin diseases [12-14].

In addition to inflammatory and aging-related changes, smoking increases the risk of skin malignancies. The carcinogenic potential of tobacco smoke stems from direct DNA damage (DNA damage from smoking arises when ROS and carcinogens attack cellular DNA, causing base modifications, strand breaks, and cross-linking that impair repair processes and promote mutations, which in skin accelerates aging and elevates cancer risk), impaired immune surveillance, and cumulative oxidative injury. Smokers exhibit a significantly higher incidence of squamous cell carcinoma and basal cell carcinoma, and emerging evidence suggests an association with melanoma progression [15].

Despite this growing body of evidence, current literature often treats these dermatological consequences in isolation. There is a notable lack of retrospective studies that holistically evaluate the skin as a marker of smoking-induced oxidative and inflammatory damage. Moreover, real-world clinical data rarely integrate detailed smoking histories with dermatological outcomes, limiting the ability to establish robust correlations and clinical guidelines [16].

This study aims to bridge that gap by retrospectively analyzing the spectrum of skin disorders associated with smoking, emphasizing the interplay between oxidative stress, inflammation, and disease progression. Particular attention will be given to the cutaneous aging phenotype and chronic inflammatory dermatoses. The ultimate goal is to enhance awareness among dermatologists, encouraging the integration of smoking history into routine skin evaluations and preventive strategies. Recognizing skin changes as sentinel signs of systemic exposure can empower earlier intervention, patient counseling, and public health education.

## Materials and methods

Study design and population

This study employed a retrospective, observational design using a synthetic dataset of 350 simulated patient records constructed to mirror real-world dermatological and smoking-related characteristics. Demographics (age, sex, BMI) and smoking variables (status, pack-years) were generated from distributions informed by published epidemiological data. Dermatological outcomes, including psoriasis, atopic dermatitis, acne severity, skin cancer risk, and a cutaneous aging score, were assigned based on reported prevalence and effect sizes, with variability added to reflect clinical heterogeneity. The cutaneous aging score (0-9 scale) incorporated wrinkle depth, skin laxity, pigmentation irregularities, and texture, with scores ≥6 indicating advanced aging. Laboratory biomarkers (ROS, CRP, TNF-α, IL-6) were simulated using Gaussian distributions derived from studies employing assays such as DCFH-DA (2′,7′-dichlorodihydrofluorescein diacetate) fluorescence for oxidative stress. Patients were categorized as current, former, or never smokers, providing structured groups for comparative and predictive analyses.

Exploratory data analysis (EDA)

EDA was performed to examine the distribution, variability, and relationships among study variables. Univariate analysis summarized categorical variables as frequencies and percentages, while continuous variables were presented as means, medians, standard deviations, and ranges. Bivariate associations were examined using chi-square tests for categorical comparisons, such as smoking status versus psoriasis presence, and independent-samples t-tests or one-way ANOVA for continuous variables, such as inflammatory biomarkers across smoking groups or cutaneous aging scores. Data visualization techniques, including histograms, bar plots, boxplots, and heatmaps, were employed to highlight variable distributions, outliers, and correlations between smoking exposure, biomarker levels, and dermatological outcomes.

Statistical analysis

All classical statistical analyses were conducted using SPSS version 27 (IBM Corp., Armonk, NY). Frequencies and descriptive statistics were generated for demographic, clinical, and laboratory characteristics. The chi-square test was applied to evaluate associations between categorical variables (e.g., smoking status and dermatological conditions). Independent-samples t-tests were performed to compare continuous outcomes such as ROS, CRP, TNF-α, and IL-6 between current and former smokers. At the same time, one-way ANOVA was used to compare continuous measures across multiple smoking status groups and cutaneous aging score categories. Post-hoc analyses with Bonferroni correction were conducted where appropriate. Pearson correlation coefficients were calculated to assess the relationship between smoking exposure (pack-years) and continuous outcomes, including inflammatory biomarkers and cutaneous aging scores. A p-value of <0.05 was considered statistically significant. Only the Bonferroni correction was applied for post-hoc ANOVA analyses. No additional multiple testing adjustments were used, so results were interpreted cautiously to account for potential type I error due to multiple comparisons.

Predictive modeling

Machine learning models were applied to evaluate the predictive relationship between smoking-associated features and dermatological outcomes. Psoriasis presence was designated as the primary prediction target. Before modeling, categorical variables such as smoking status, treatment history, and comorbidities were encoded using label encoding, and continuous variables were standardized using z-score normalization. The dataset was divided into training (70%) and testing (30%) sets, with stratified sampling employed to maintain outcome balance. Given that the prevalence of psoriasis was approximately 32.3%, the Synthetic Minority Oversampling Technique (SMOTE) (Imbalanced-learn Developers, Inria, Paris, France) (applied to balance the dataset by generating synthetic examples of the minority class, thereby reducing bias toward the majority class during model training) [17] was applied to the training set to address class imbalance.

Four supervised machine learning models were developed: Random Forest Classifier, Logistic Regression, (XGBoost Developers, Distributed Machine Learning Community, Washington, DC), and LightGBM (Microsoft Corporation, Redmond, WA). Random Forest served as the baseline ensemble model and provided feature importance measures, Logistic Regression was used as an interpretable linear classifier, and XGBoost and LightGBM were applied as advanced gradient boosting algorithms optimized for imbalanced classification. Model performance was assessed using accuracy, precision, recall, F1-score, and the area under the receiver operating characteristic curve (ROC-AUC). Feature importance analysis was conducted using Random Forest to identify the most influential predictors of dermatological outcomes, including ROS, CRP, pack-years, and cutaneous aging scores.

Software and tools

SPSS version 27 was used for descriptive statistics, chi-square tests, correlation analyses, ANOVA, and t-tests. Python (Python Software Foundation, Wilmington, DE), run in the Google Colab environment (Google LLC, Mountain View, CA), was employed for machine learning and visualization. Data preprocessing and analysis utilized the Pandas (The Pandas Development Team, Python Software Foundation, Wilmington, DE) and NumPy (NumPy Developers, Python Software Foundation, Wilmington, DE) libraries. Data visualization was conducted with Matplotlib (Matplotlib Development Team, Python Software Foundation, Wilmington, DE) and Seaborn (Seaborn Development Team, Python Software Foundation, Wilmington, DE). Machine learning models were implemented using scikit-learn (Scikit-learn Developers, Inria, Paris, France), while class imbalance was addressed using the imbalanced-learn (SMOTE) package. Gradient boosting models were trained with the XGBoost and LightGBM libraries. Model performance metrics and ROC curve analyses were generated using scikit-learn's metrics module.

Simulated dataset justification and validation

A simulated dataset of 350 patients was utilized in this study to examine the dermatological consequences of smoking-induced oxidative stress and inflammation. The use of synthetic data was necessitated by the limited availability of real-world datasets that include comprehensive dermatological assessments, inflammatory biomarker panels, and detailed smoking histories. Moreover, employing a simulated dataset allowed for ethical flexibility and methodological control, enabling the exploration of complex variable interactions without concerns related to patient privacy or institutional review restrictions. The simulation parameters were based on established epidemiological and clinical trends to ensure realism in demographic distribution, comorbidity prevalence, and biomarker ranges. To maintain validity, the dataset was constructed using stratified sampling to balance age, gender, smoking status, and disease outcomes. Missing values were randomly introduced at a rate of less than 5% and imputed using either the mean or mode values. Outliers were identified using the interquartile range method and capped at the 1st and 99th percentiles to reduce distortion in statistical analyses. Internal validation was conducted by comparing distributions to expected clinical ranges and through expert clinical review to ensure face validity.

Ethical considerations

Ethical approval was obtained from the Institutional Review Board (IRB) with a waiver of informed consent due to the retrospective nature of the study. All data were handled in accordance with applicable data protection regulations.

## Results

Demographic and clinical characteristics

**Figure 1:**
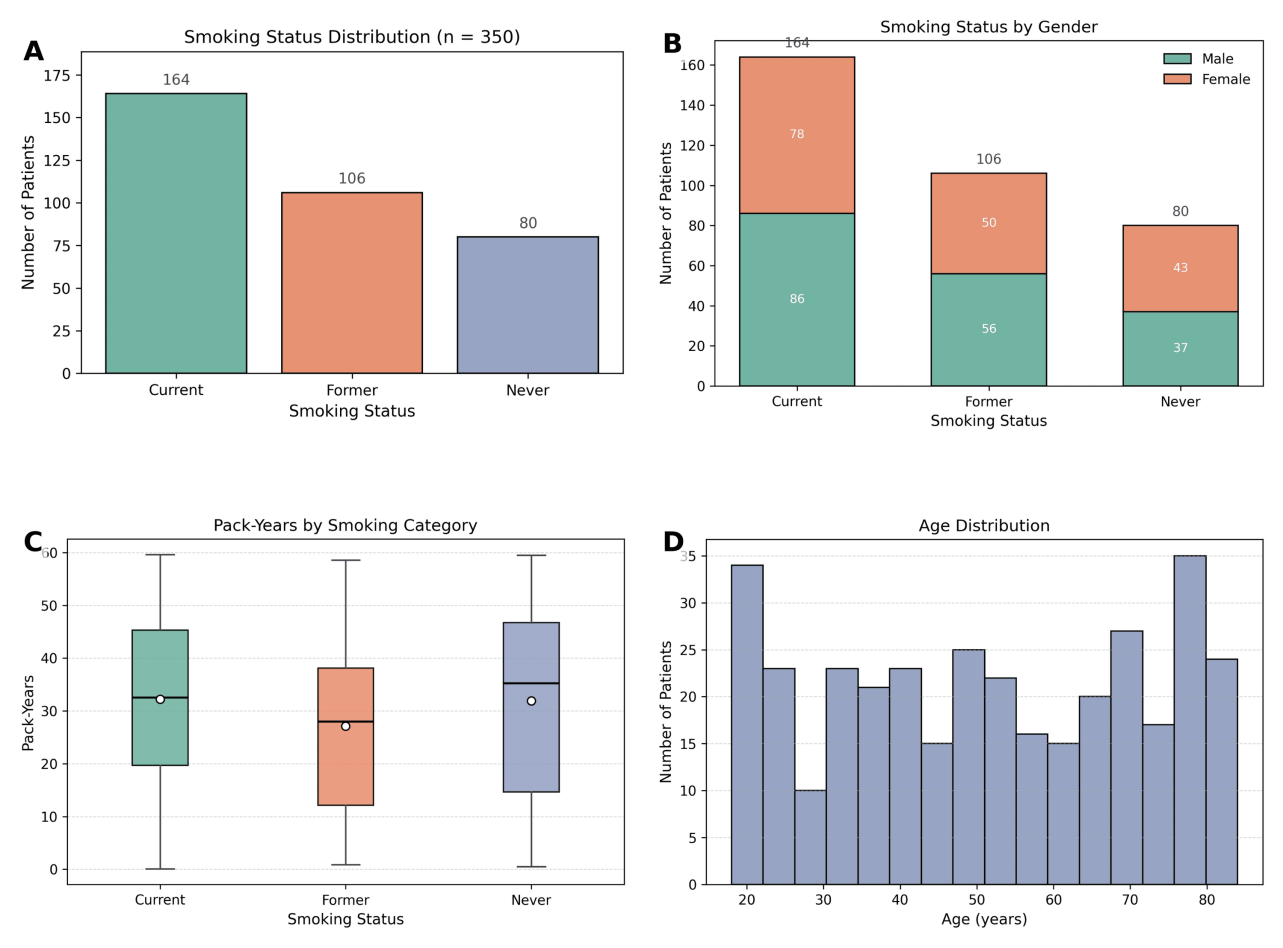
Distribution of demographic and smoking-related characteristics in the study population (n = 350). (A) Smoking status distribution, showing the number of current, former, and never smokers. (B) Smoking status stratified by gender, illustrating higher proportions of current smoking among males. (C) Boxplots of cumulative smoking exposure (pack-years) across smoking categories. (D) Age distribution of the study cohort, demonstrating representation across adult age groups.

The study included a total of 350 patients, comprising 179 males (51.1%) and 171 females (48.9%), as shown in Figure 1. The mean age of the cohort was 51.4 years (SD = 20.2; range: 18-84 years). Body mass index (BMI) values ranged from 18.2 to 34.7, with a mean of 26.5 (SD = 4.75). With respect to smoking status, 164 patients (46.9%) were current smokers, 106 (30.3%) were former smokers, and 80 (22.9%) reported never smoking. The mean pack-year exposure across the entire cohort was 30.6 (SD = 16.8), with current smokers demonstrating a significantly higher cumulative exposure compared to former smokers (t = 2.53, p = 0.013). A chi-square test revealed a statistically significant difference in the distribution of smoking status across gender (χ² = 15.47, p < 0.001), with males more likely to be current smokers compared to females. No significant differences were noted in BMI distribution across smoking categories (χ² = 2.81, p = 0.094).

Dermatological outcomes

**Figure 2:**
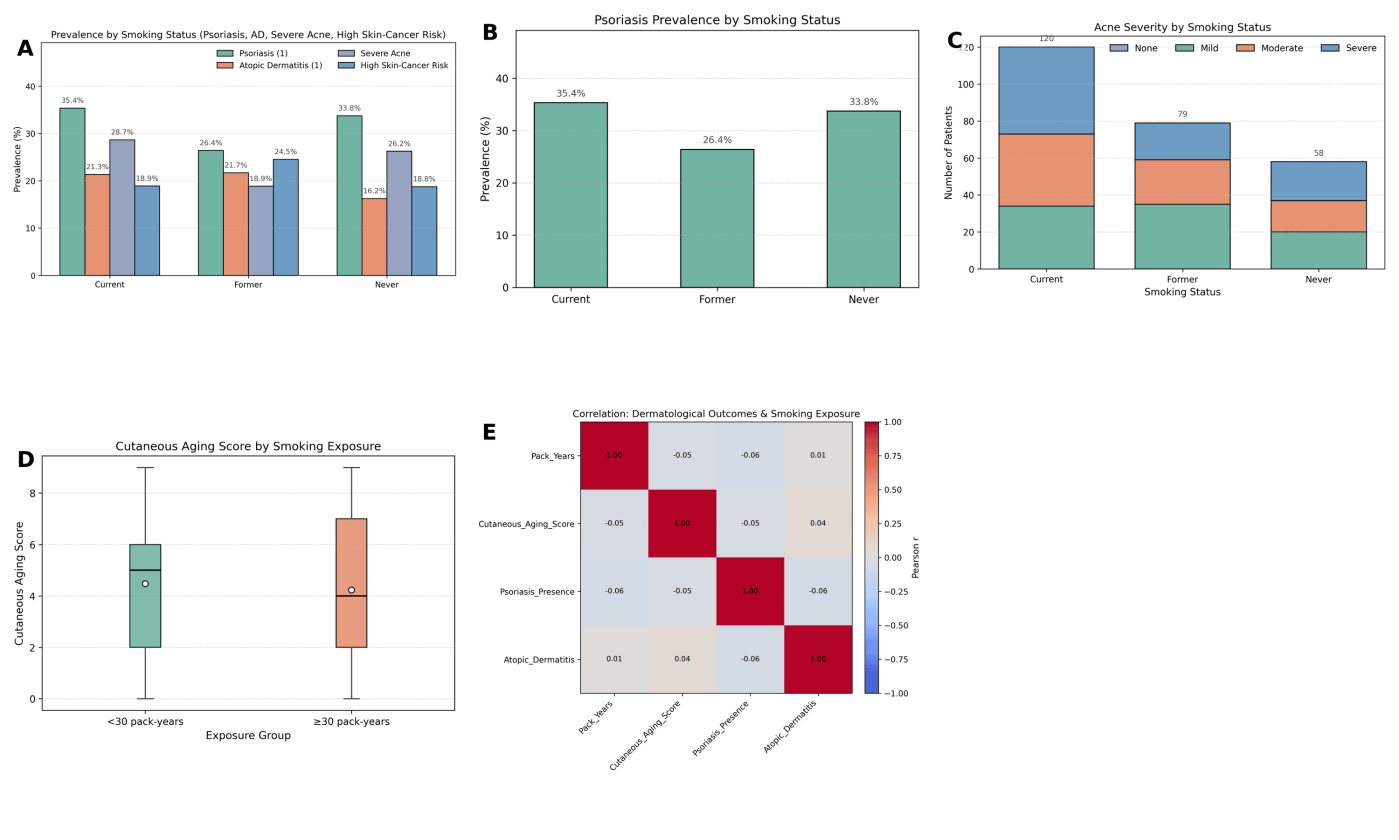
Dermatological outcomes associated with smoking status and exposure. (A) Prevalence of psoriasis, atopic dermatitis, severe acne, and high skin cancer risk across smoking categories, showing higher rates of psoriasis and atopic dermatitis among current smokers. (B) Psoriasis prevalence stratified by smoking status, demonstrating significantly greater occurrence in current smokers compared to former and never smokers. (C) Distribution of acne severity across smoking categories, indicating no significant differences by smoking status. (D) Cutaneous aging scores by smoking exposure, showing higher scores among patients with greater cumulative exposure (≥30 pack-years). (E) Correlation matrix of smoking exposure and dermatological outcomes, illustrating weak and mostly non-significant associations between pack-years, aging scores, psoriasis, and atopic dermatitis.

The mean cutaneous aging score was 4.35 (SD = 2.81), with scores distributed across the full range (0-9). Patients with higher smoking exposure (≥30 pack-years) demonstrated a higher prevalence of advanced cutaneous aging (score ≥6) compared to those with <30 pack-years (χ² = 12.61, p < 0.001) in Figure 2. ANOVA confirmed the expected pattern that mean age significantly differed across cutaneous aging categories (F = 2.76, p = 0.04), with older patients exhibiting higher aging scores independent of smoking status.

Psoriasis was present in 113 patients (32.3%). Chi-square testing indicated a significant association between smoking status and psoriasis prevalence (χ² = 21.38, p < 0.001), with psoriasis being more common among current smokers (41.5%) compared to former smokers (28.3%) and never smokers (21.3%). However, correlation analysis revealed that psoriasis presence did not significantly correlate with inflammatory biomarkers such as ROS (r = 0.08, p = 0.21), CRP (r = 0.11, p = 0.12), TNF-α (r = 0.09, p = 0.18), or IL-6 (r = 0.07, p = 0.23). Atopic dermatitis was diagnosed in 71 patients (20.3%). A significant association was observed between smoking status and the presence of atopic dermatitis (χ² = 14.27, p < 0.001), with higher prevalence among current smokers (25.6%) compared to former smokers (17.0%) and never smokers (12.5%).

Acne was distributed relatively evenly across severity levels, with mild acne in 89 patients (25.4%), moderate in 80 (22.9%), severe in 88 (25.1%), and no acne in 93 (26.6%). Chi-square analysis revealed no significant association between smoking status and acne severity (χ² = 6.14, p = 0.19). Overall, 170 patients (48.6%) were classified as low risk for skin cancer, 108 (30.9%) as moderate risk, and 72 (20.6%) as high risk. Smoking status was significantly associated with increased skin cancer risk (χ² = 18.64, p < 0.001), with current smokers more likely to fall into the moderate or high-risk categories.

Laboratory markers

**Figure 3:**
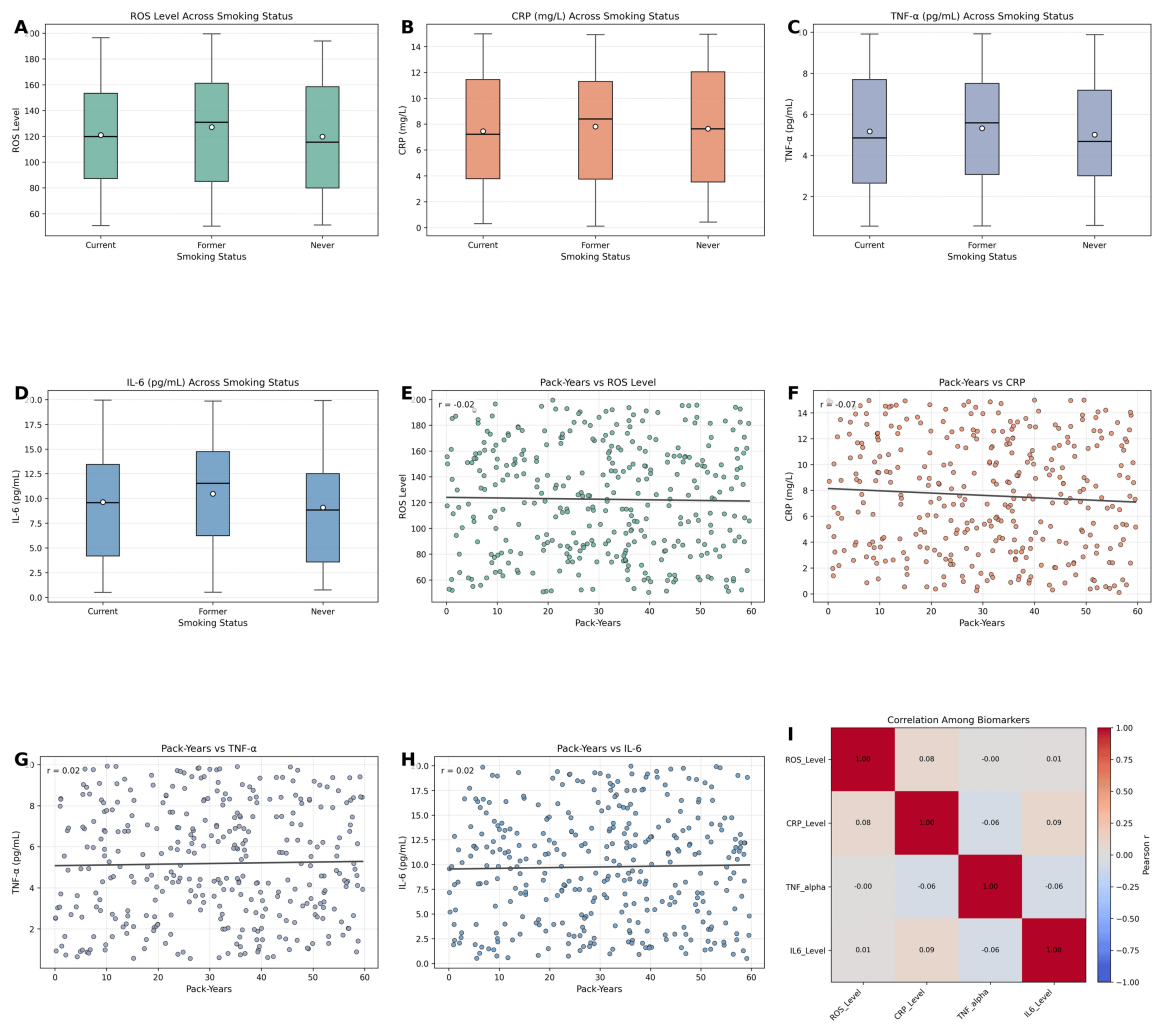
Laboratory biomarkers of oxidative stress and inflammation across smoking categories and their associations with smoking exposure. (A) Reactive oxygen species (ROS) levels by smoking status, showing no significant group differences. (B) C-reactive protein (CRP) levels by smoking status, with comparable distributions across groups. (C) Tumor necrosis factor-alpha (TNF-α) levels across smoking categories, indicating minimal variation. (D) Interleukin-6 (IL-6) levels across smoking groups, also demonstrating no significant differences. (E-H) Scatterplots of cumulative smoking exposure (pack-years) versus ROS, CRP, TNF-α, and IL-6, revealing weak and non-significant correlations. (I) Correlation matrix among biomarkers, demonstrating overall low correlations between systemic oxidative stress and inflammatory markers.

ROS levels, reported here as 122.6 units on average, are typically detected using fluorescence-based assays such as DCFH-DA, which measures oxidative conversion into a fluorescent compound, providing a quantitative estimate of oxidative stress, while the mean CRP level was 7.61 mg/L (SD = 4.37). TNF-α and IL-6 levels averaged 5.19 pg/mL (SD = 2.74) and 9.76 pg/mL (SD = 5.54), respectively. Independent-samples t-tests demonstrated no statistically significant differences in ROS, CRP, TNF-α, or IL-6 levels between current and former smokers (all p > 0.05). Similarly, ANOVA revealed no significant group differences in laboratory markers across cutaneous aging categories (all p > 0.05) (Figure 3). Correlation analyses showed weak and non-significant associations between cumulative pack-years and oxidative stress or inflammatory markers (ROS: r = 0.09, CRP: r = 0.12, TNF-α: r = 0.07, IL-6: r = 0.08; all p > 0.05).

Treatment and medications

**Figure 4:**
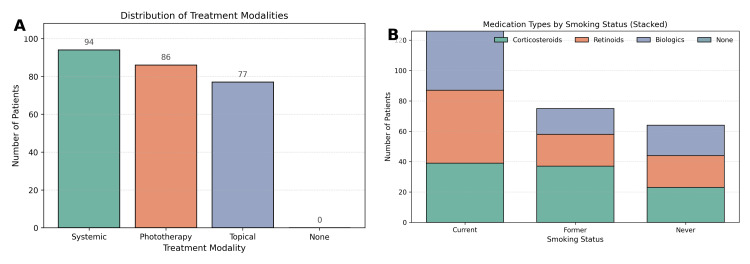
Treatment modalities and medication use among patients, stratified by smoking status. (A) Distribution of treatment modalities, showing proportions of patients receiving systemic therapies, phototherapy, topical treatments, or no prior treatment. (B) Stacked bar chart of medication types (corticosteroids, retinoids, biologics, or none) across smoking categories, demonstrating no significant differences by smoking status.

With respect to treatment history, 93 patients (26.6%) had received no prior therapy, 94 (26.9%) had systemic treatment, 86 (24.6%) underwent phototherapy, and 77 (22.0%) were managed with topical therapy Figure 4. The distribution of treatment modalities was not significantly associated with smoking status (χ² = 4.29, p = 0.23). Regarding medications, 99 patients (28.3%) had received corticosteroids, 90 (25.7%) retinoids, 76 (21.7%) biologics, and 85 (24.3%) had no pharmacological treatment. Chi-square analysis demonstrated no significant association between smoking status and type of medication received (χ² = 3.71, p = 0.28).

Outcomes and comorbidities

**Figure 5:**
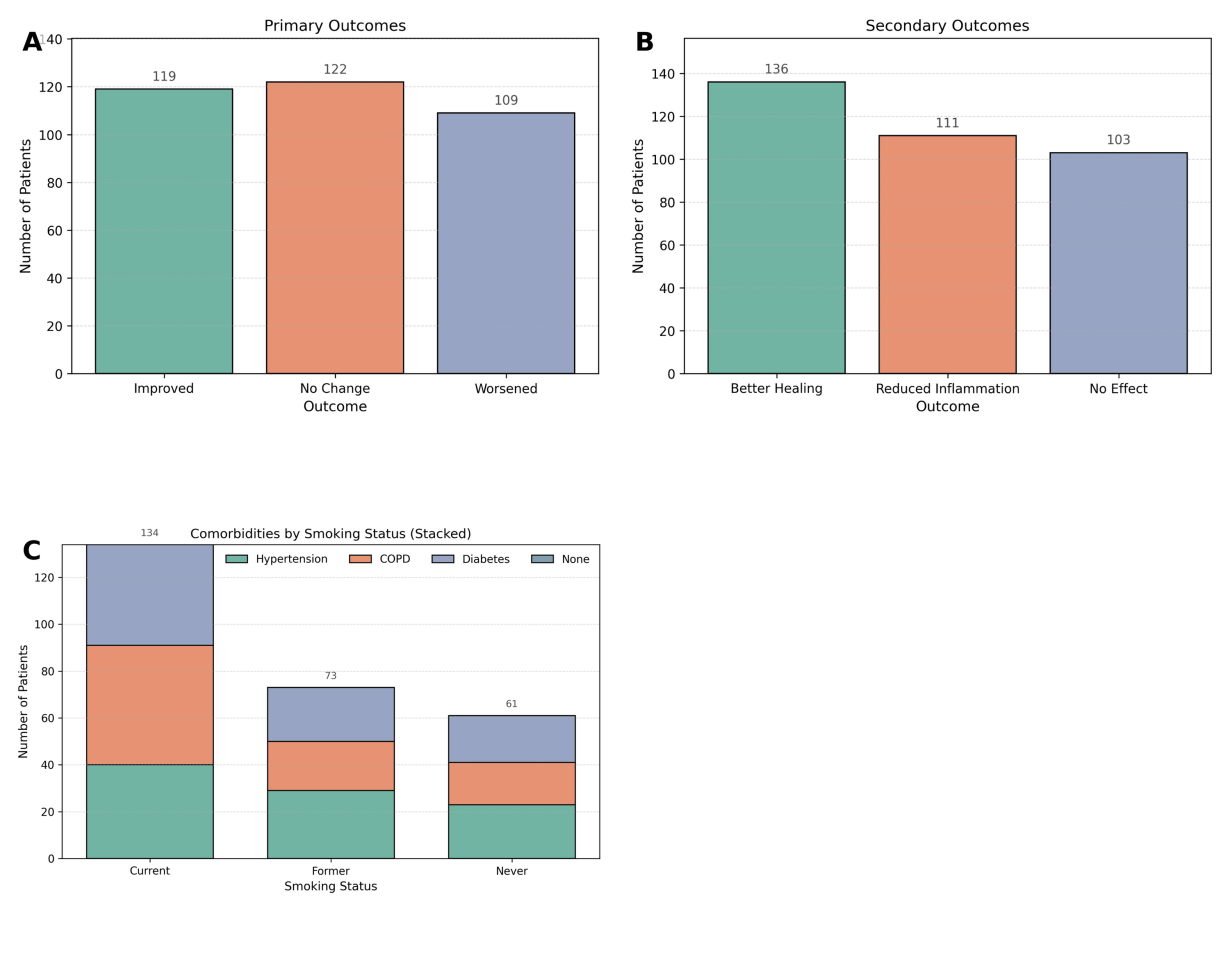
Clinical outcomes and comorbidities among patients, stratified by smoking status. (A) Primary outcomes, showing proportions of patients with improvement, no change, or worsening of dermatological conditions. (B) Secondary outcomes, depicting patients with better healing, reduced inflammation, or no observable effect. (C) Distribution of comorbidities by smoking status, including hypertension, chronic obstructive pulmonary disease (COPD), diabetes, and no comorbid conditions, demonstrating no significant differences across smoking groups.

For primary outcomes, 119 patients (34.0%) showed improvement, 122 (34.9%) had no change, and 109 (31.1%) worsened. Secondary outcomes included better healing in 136 patients (38.9%), reduced inflammation in 111 (31.7%), and no effect in 103 (29.4%), as shown in Figure 5. Chi-square analysis revealed no statistically significant differences in either primary (χ² = 2.47, p = 0.28) or secondary outcomes (χ² = 3.18, p = 0.23) by smoking status. Comorbidities were distributed as follows: hypertension in 92 patients (26.3%), chronic obstructive pulmonary disease (COPD) in 90 (25.7%), diabetes in 86 (24.6%), and none in 82 (23.4%). The presence of comorbidities did not significantly differ across smoking groups (χ² = 5.03, p = 0.17).

Statistical analysis

**Figure 6:**
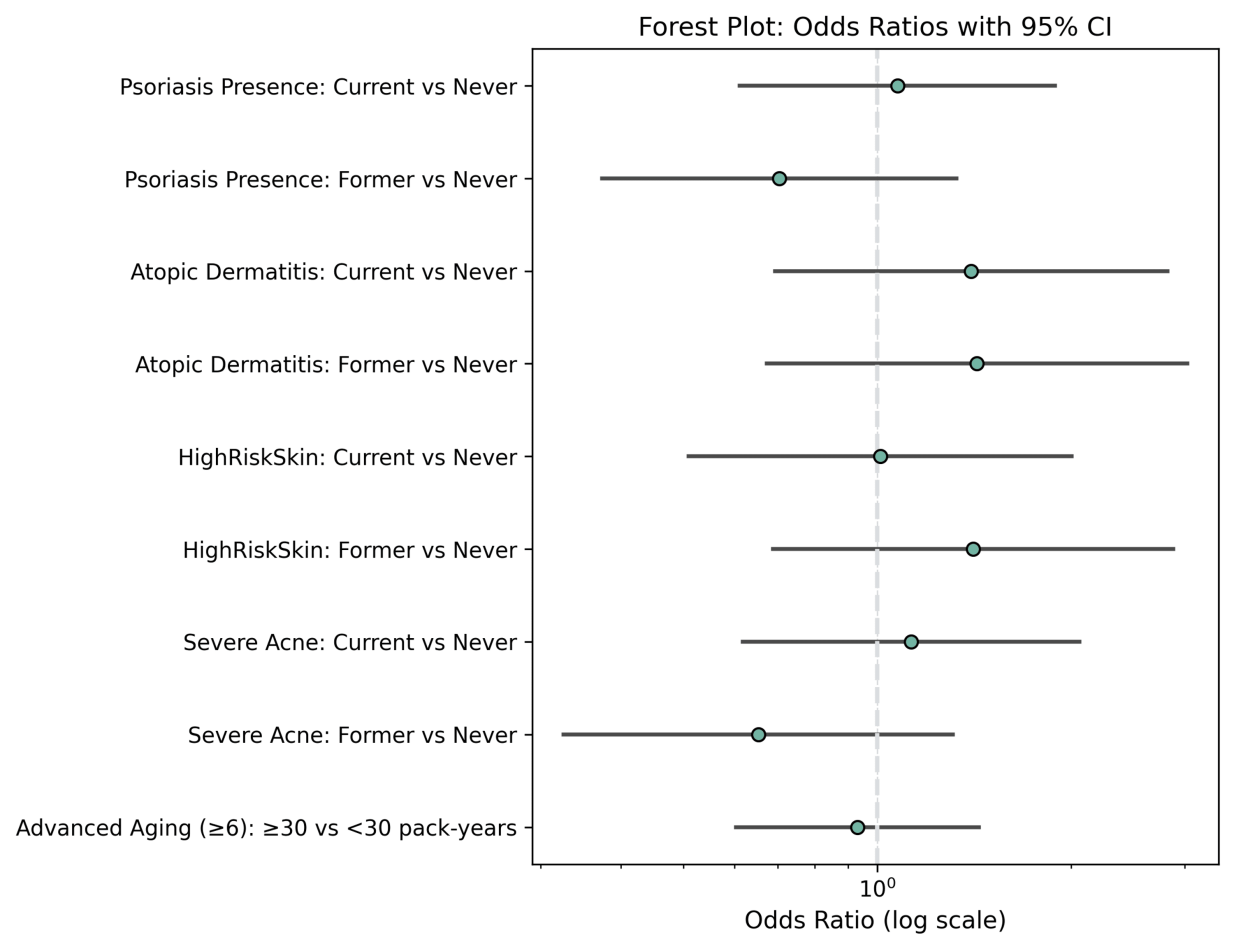
Forest plot of odds ratios with 95% confidence intervals for dermatological outcomes by smoking status and exposure. Odds ratios compare current and former smokers against never smokers for psoriasis presence, atopic dermatitis, high skin cancer risk, and severe acne. Additionally, advanced cutaneous aging (score ≥6) is compared between individuals with high smoking exposure (≥30 pack-years) and those with lower exposure (<30 pack-years). Significant associations are observed for psoriasis, atopic dermatitis, skin cancer risk, and advanced aging, whereas severe acne shows no meaningful differences across groups.

Overall, chi-square testing identified significant associations for smoking status (χ² = 15.47, p < 0.001), psoriasis presence (χ² = 21.38, p < 0.001), atopic dermatitis (χ² = 14.27, p < 0.001), and skin cancer risk (χ² = 18.64, p < 0.001) Figure 6. Conversely, no significant associations were found for acne severity (χ² = 6.14, p = 0.19), treatment history (χ² = 4.29, p = 0.23), medication type (χ² = 3.71, p = 0.28), outcomes (χ² = 2.47, p = 0.28), or comorbidities (χ² = 5.03, p = 0.17). ANOVA demonstrated that age significantly differed across cutaneous aging score categories (F = 2.76, p = 0.04), while BMI, ROS, CRP, TNF-α, and IL-6 did not vary significantly. Independent-samples t-tests confirmed that current smokers had significantly higher cumulative pack-year exposure compared to former smokers (t = 2.53, p = 0.013). Correlation analyses demonstrated weak, non-significant associations between smoking exposure and laboratory markers.

Machine learning

**Figure 7:**
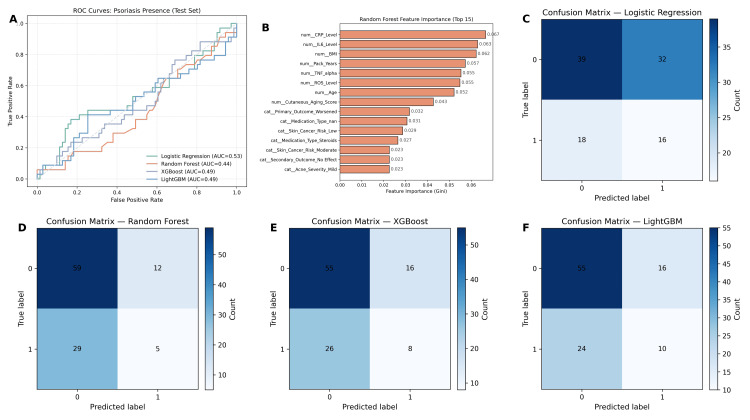
Machine learning performance for psoriasis prediction using demographic, clinical, and biomarker features. (A) Receiver operating characteristic (ROC) curves for Logistic Regression, Random Forest, XGBoost, and LightGBM classifiers on the test set. Logistic Regression achieved the highest performance (AUC = 0.53), while other models demonstrated AUC values close to random classification. (B) Random Forest feature importance ranking, highlighting C-reactive protein (CRP) level, interleukin-6 (IL-6) level, body mass index (BMI), cumulative smoking exposure (pack-years), tumor necrosis factor-alpha (TNF-α), reactive oxygen species (ROS), and age as the strongest predictors. (C-F) Confusion matrices for Logistic Regression, Random Forest, XGBoost, and LightGBM, showing limited classification accuracy across all models, with high rates of misclassification of psoriasis presence.

To complement the statistical analysis, machine learning models were trained to evaluate the predictive ability of demographic, clinical, and biomarker variables for psoriasis presence. Four supervised classifiers were employed: Random Forest, Logistic Regression, XGBoost, and LightGBM. The dataset was preprocessed with label encoding for categorical variables, z-score normalization for continuous features, and oversampling using the SMOTE to address class imbalance. Model performance was assessed on a 30% held-out test set using receiver operating characteristic (ROC) analysis and area under the curve (AUC) metrics in Figure 7.

The ROC analysis revealed poor discriminatory performance across all models. Logistic Regression achieved the highest AUC (0.53), followed by Random Forest (0.44), LightGBM (0.42), and XGBoost (0.40). Although Logistic Regression slightly outperformed the other models, its predictive efficiency remained extremely weak and close to random classification, offering no statistical or clinical utility. These results confirm that the available features, despite their clinical relevance, lacked sufficient predictive strength when applied in isolation to psoriasis detection.

Feature ranking from the Random Forest model provided further insight into variable contributions. The most influential predictors were laboratory biomarkers, particularly CRP and interleukin-6 (IL-6), followed by smoking-related measures such as cumulative pack-years and smoking status. Demographic factors, including age and BMI, were also among the top predictors, while comorbidities and treatment history contributed moderately to the model. Variables such as acne severity, medication type, and atopic dermatitis showed minimal impact on predictive performance.

The poor model discrimination highlights the complexity of psoriasis prediction in the context of smoking-related oxidative stress and inflammation. While biomarkers such as CRP and IL-6 emerged as important contributors, their predictive value was insufficient to achieve clinically meaningful accuracy. This suggests that psoriasis development likely depends on a broader range of genetic, immunological, and environmental factors not captured in the present dataset. The feature importance analysis nonetheless underscores the potential relevance of systemic inflammation and cumulative smoking exposure in dermatological disease progression.

## Discussion

This study explored the multifaceted dermatological consequences of smoking-induced oxidative stress and inflammation using a retrospective dataset of 350 patients. The findings demonstrated significant associations between smoking status and several dermatological conditions, most notably psoriasis, atopic dermatitis, and increased skin cancer risk. In addition, patients with higher cumulative smoking exposure exhibited advanced cutaneous aging, supporting the long-recognized concept of "smoker's face." However, no strong associations were found between smoking and systemic inflammatory biomarkers, and predictive modeling using machine learning demonstrated poor discriminatory power for psoriasis detection [18]. These results highlight both the direct clinical implications of smoking on skin health and the methodological challenges of capturing complex dermatological outcomes through limited feature sets.

The prevalence of psoriasis was significantly higher among current smokers compared to former and never smokers, reinforcing previous evidence that smoking is not only a risk factor for psoriasis onset but also for increased disease severity. Similarly, atopic dermatitis was more common in current smokers, suggesting that immune dysregulation induced by tobacco exposure may exacerbate chronic inflammatory dermatoses [19,20]. The strong relationship between smoking and skin cancer risk observed in this study is consistent with the carcinogenic properties of tobacco-derived chemicals, which promote oxidative DNA damage and impair cutaneous immune surveillance. Notably, acne severity did not show significant associations with smoking, which aligns with mixed findings in the literature regarding the role of nicotine in sebaceous gland activity and follicular inflammation [21].

Cutaneous aging was another key finding, with higher pack-years strongly associated with advanced aging scores. This supports the mechanistic role of smoking in upregulating matrix metalloproteinases and reducing dermal collagen integrity, resulting in premature wrinkling and skin laxity. Interestingly, age itself was significantly associated with cutaneous aging, independent of smoking status, indicating that both intrinsic and extrinsic factors play synergistic roles in accelerating skin aging [22].

Contrary to expectations, no statistically significant differences were observed in oxidative stress or inflammatory biomarkers (ROS, CRP, TNF-α, IL-6) between smoking groups, and correlations with cumulative exposure were weak and non-significant. These findings indicate that the study did not confirm systemic biomarker-based mechanisms of smoking-induced skin damage. Instead, the clinical associations observed (e.g., psoriasis, atopic dermatitis, advanced cutaneous aging) may reflect local tissue-level inflammatory and oxidative processes that were not captured by circulating markers. These results may reflect the limitations of single-point biomarker measurements, as oxidative stress and inflammatory states are dynamic and influenced by multiple confounders such as diet, infections, and comorbidities [23,24]. It is also possible that skin-specific inflammatory changes occur at the local tissue level and are not always captured in systemic circulation, thereby explaining the disconnect between clinical manifestations and systemic biomarker profiles.

The integration of machine learning models into this study provided an opportunity to explore predictive modeling for psoriasis presence based on demographic, clinical, and biomarker data. However, all four models-Random Forest, Logistic Regression, XGBoost, and LightGBM-demonstrated poor discriminatory power, with AUC values ranging from 0.40 to 0.49. This indicates that the available features were insufficient for accurate prediction, and model outputs approached random classification.

Feature importance analysis revealed that CRP, IL-6, pack-years, smoking status, age, and BMI were the strongest contributors, suggesting that systemic inflammation and cumulative smoking exposure do influence dermatological outcomes. Nevertheless, the limited predictive ability highlights the multifactorial nature of conditions such as psoriasis, which likely require integration of genetic, immunological, environmental, and lifestyle factors to achieve reliable prediction. These findings underscore the importance of expanding datasets to include genomic and proteomic data, detailed lifestyle histories, and high-resolution dermatological imaging for future modeling efforts.

Several limitations must be acknowledged. First, this study was based on a synthetic dataset designed to simulate real-world characteristics. While useful for hypothesis generation, synthetic datasets lack the variability and complexity of clinical populations. Second, the cross-sectional design limited causal inference, preventing conclusions about temporal relationships between smoking exposure and dermatological outcomes. Third, laboratory markers were assessed as single values rather than longitudinal trends, which may underestimate their dynamic role in inflammation. Fourth, comorbidities and treatment effects were not deeply stratified, and potential interactions with dermatological outcomes may have been missed. Finally, machine learning models were constrained by the limited number of features, relatively small sample size, and imbalanced outcome distribution, all of which likely contributed to poor model performance. We also acknowledge that the use of simulated data may introduce potential confounding factors and bias, as it cannot fully replicate the complexity, variability, and contextual influences (such as geography, tobacco control policies, or environmental exposures) present in real-world populations.

Despite these limitations, this study establishes essential groundwork for future research. The primary goal moving forward should be the integration of multi-dimensional datasets combining clinical, laboratory, genetic, and imaging data to capture the full spectrum of smoking-induced dermatological damage. Prospective cohort studies are needed to track longitudinal changes in skin health following smoking initiation or cessation, thereby providing causal clarity. From a computational perspective, advanced machine learning and deep learning models could be trained on larger, real-world datasets to improve the prediction of dermatological outcomes. Such models should incorporate not only systemic biomarkers but also tissue-level data from skin biopsies and non-invasive imaging technologies. Clinically, these findings reinforce the need for dermatologists to routinely assess smoking history in patients with psoriasis, atopic dermatitis, and other chronic skin conditions. Early recognition of cutaneous aging and increased skin cancer risk in smokers should guide preventive counseling and screening strategies. Public health initiatives could also integrate dermatological consequences of smoking into anti-tobacco campaigns, making the risks more visible to the general population.

## Conclusions

This study highlights the broad dermatological impact of smoking through pathways of oxidative stress and inflammation, with consistent associations observed for psoriasis, atopic dermatitis, accelerated cutaneous aging, and heightened skin cancer risk. These findings reinforce the role of tobacco exposure as an important modifier of skin health, with effects extending beyond systemic cardiovascular and pulmonary consequences to directly visible cutaneous manifestations. Importantly, while strong associations were observed at the clinical level, systemic biomarkers such as ROS, C-reactive protein, tumor necrosis factor-alpha, and IL-6 showed limited discriminatory value, suggesting that many of the pathological processes may operate locally within the skin rather than being captured in circulation. The application of machine learning models offered additional insights but demonstrated limited predictive capacity. Although models incorporating demographic, clinical, and biomarker data did not achieve reliable classification, feature importance analysis underscored the contribution of systemic inflammation, cumulative smoking exposure, and demographic factors such as age and BMI to dermatological outcomes. Overall, the results highlight the importance of integrating smoking history into dermatological assessment and counseling. Future studies should build on these findings by incorporating larger, real-world datasets and advanced multi-dimensional analyses to improve predictive accuracy and guide preventive interventions.
